# Exosomal MicroRNAs as Drivers of Desmoplasia and Treatment Resistance in Breast Cancer: Mechanisms, Biomarker Potential, and Therapeutic Opportunities

**DOI:** 10.3390/biom16050682

**Published:** 2026-05-05

**Authors:** Jun Chung, Young Hwa Soung

**Affiliations:** Department of Pathology, Renaissance School of Medicine, Stony Brook University, Stony Brook, NY 11794, USA; jun.chung@stonybrookmedicine.edu

**Keywords:** breast cancer, exosomes, microRNA, desmoplasia, tumor microenvironment, cancer-associated fibroblasts, chemoresistance, endocrine resistance, miR-181 family, liquid biopsy, mechanotransduction, triple-negative breast cancer

## Abstract

Exosomal microRNAs (miRNAs) are key mediators of intercellular communication in the breast cancer tumor microenvironment (TME), facilitating bidirectional signaling between malignant cells and the desmoplastic stroma. This review explores current evidence on their dual roles as drivers of stromal remodeling and as circulating biomarkers of therapeutic resistance across major breast cancer subtypes, including triple-negative breast cancer (TNBC), hormone receptor-positive (ER+/PR+) disease, and HER2-amplified tumors. We outline how miR-9, miR-21, and miR-181 family members promote cancer-associated fibroblast (CAF) activation, increase extracellular matrix (ECM) stiffness, and sustain a reverse Warburg phenotype. We then detail subtype-specific resistance mechanisms: miR-181 family members suppress BCLAF1 to block doxorubicin-induced apoptosis; miR-221/222 downregulates ESR1 and p27Kip1 to confer tamoxifen resistance; miR-155 impairs homologous recombination in TNBC; and miR-1246 sustains PI3K/AKT signaling in HER2-positive disease. We also evaluate circulating exosomal miRNA panels as liquid biopsy tools for predicting chemotherapy response and tracking resistance emergence. Finally, we discuss therapeutic strategies including antagomirs, miRNA replacement therapy and engineered exosome platforms, and address key challenges such as assay standardization and regulatory hurdles, that must be overcome for clinical translation.

## 1. Introduction

Breast cancer remains the most commonly diagnosed malignancy among women globally, accounting for an estimated 2.3 million new cases and approximately 685,000 deaths each year [1]. Although therapeutic options have expanded considerably, with the introduction of targeted kinase inhibitors, CDK4/6 inhibitors, immune checkpoint blockade, and antibody-drug conjugates, a substantial number of patients still develop either primary or acquired resistance to treatment. This challenge is particularly pronounced in triple-negative breast cancer (TNBC), a subtype defined by the absence of estrogen receptor (ER), progesterone receptor (PR), and HER2 amplification. In the absence of established molecular targets, cytotoxic chemotherapy remains the backbone of systemic therapy, contributing to persistently poor outcomes in this patient population [2]. An often-underappreciated contributor to therapeutic failure is the tumor microenvironment (TME), a complex ecosystem comprising stromal fibroblasts, immune cells, vascular elements, and the extracellular matrix (ECM). In aggressive breast cancers, especially TNBC, the TME undergoes desmoplasia, a reactive fibrotic process driven by cancer-associated fibroblasts (CAFs) that leads to excessive collagen deposition and increased matrix stiffness [3]. This rigid stroma not only creates a physical barrier to drug penetration, through elevated interstitial fluid pressure and dense ECM architecture, but also provides biochemical and mechanical signals that enhance tumor cell survival and facilitate metastatic spread [4].

Exosomes—nanoscale extracellular vesicles (30–150 nm) that transport bioactive molecular cargo, particularly microRNAs (miRNAs)—are at the core of the intercellular communication network that sustains desmoplasia. MiRNAs are short, around 18–22 nucleotide non-coding RNAs that regulate gene expression post-transcriptionally by binding to complementary sequences within the 3′-untranslated regions (3′-UTRs) of target messenger RNAs, leading to translational suppression or mRNA degradation [5,6]. Importantly, packaging within the exosomal lipid bilayer protects miRNAs from degradation by circulating RNases, granting them exceptional stability in biofluids such as plasma, serum, urine, and breast milk. This inherent resilience, distinct from the vulnerability of unprotected cell-free miRNAs, positions exosomal miRNAs as robust and accessible analytes for liquid biopsy applications [7].

Exosomal miRNA signaling in breast cancer operates through two interconnected mechanisms that reinforce disease progression and treatment resistance. First, tumor-derived exosomal miRNAs, including miR-9, miR-155, members of the miR-181 family, miR-221/222, and miR-21, actively reprogram quiescent stromal fibroblasts into activated cancer-associated fibroblasts (CAFs), thereby initiating and sustaining desmoplasia. In parallel, CAF-derived exosomal miRNAs, particularly those from the miR-181 family and miR-221/222, are transferred back to cancer cells. Once internalized, these miRNAs potentiate survival signaling through pathways such as PTEN/PI3K/AKT and contribute to resistance against chemotherapy, endocrine therapy, and HER2-targeted agents [8,9]. The distinct contributions of individual miR-181 isoforms—miR-181a, miR-181b-5p, and miR-181d-5p—are discussed in Section 3 and Section 4. This bidirectional exchange establishes a self-perpetuating loop that couples stromal remodeling with acquired therapeutic resistance. Second, because the composition of exosomal miRNAs closely reflects the functional state of both tumor and stromal compartments in real time, circulating exosomal miRNA profiles offer a dynamic, non-invasive window into tumor biology. This enables serial monitoring of disease status and treatment response, a capability not feasible with conventional tissue biopsies and one that holds significant promise for liquid biopsy applications.

This review consolidates current mechanistic and clinical evidence on the role of exosomal miRNAs in breast cancer desmoplasia and treatment resistance. We focus on five key areas: (1) the processes governing exosome biogenesis and selective packaging of miRNA cargo; (2) the contribution of exosomal miRNAs to CAF activation and extracellular matrix remodeling across breast cancer subtypes; (3) major resistance pathways driven by exosomal miRNAs and their convergence on central signaling hubs; (4) clinical data supporting the use of circulating exosomal miRNA panels as non-invasive biomarkers; and (5) emerging therapeutic approaches aimed at disrupting this axis, along with the translational hurdles that remain.

## 2. Exosome Biogenesis and Selective miRNA Cargo Loading

### 2.1. MVB Formation and Exosome Secretion

Exosomes originate through the endosomal pathway. After endocytosis, early endosomes mature into multivesicular bodies (MVBs) as the limiting endosomal membrane buds inward to generate intraluminal vesicles (ILVs). When MVBs fuse with the plasma membrane, these ILVs are released into the extracellular space as exosomes [10]. Two primary biogenesis pathways operate in breast cancer cells. The canonical ESCRT-dependent route, mediated by components such as HRS, TSG101, and ALIX, sorts ubiquitinated cargo into ILVs. An alternative, ESCRT-independent pathway relies on ceramide generation by neutral sphingomyelinase 2 (nSMase2) to promote membrane curvature and ILV formation; this process can be pharmacologically inhibited by GW4869 [11]. Rab GTPases—notably Rab27a and Rab27b—regulate the docking and fusion of MVBs with the plasma membrane, thereby controlling the rate of exosome release [12].

In desmoplastic breast cancer, both tumor cells and activated CAFs show markedly elevated exosome secretion compared to normal mammary fibroblasts. This increase is driven HIF-1α stabilization under hypoxic conditions, and the composition of CAF-derived exosomal cargo evolves as desmoplasia progresses [13]. Importantly, both the quantity and the miRNA composition of secreted exosomes evolve as desmoplasia progresses [14].

A note on terminology: per MISEV2023 guidelines, “exosome” strictly denotes small EVs (extracellular vesicles) of confirmed endosomal/MVB origin, whereas most studies cited here used generic isolation methods that do not permit definitive subtype assignment. We retain “exosome” throughout to match the terminology of the cited primary literature, with the understanding that these vesicles more precisely represent small EVs enriched for endosomal markers.

### 2.2. Selective miRNA Sorting into Exosomes

Exosomal miRNA content is not merely a passive reflection of the cellular miRNome—specific molecular mechanisms actively govern the preferential packaging of distinct miRNA species. In desmoplastic breast cancer, selective cargo loading is a critical determinant of which oncomiRs are amplified within the CAF-tumor signaling loop. Among the best-characterized sorting regulators, the RNA-binding protein hnRNPA2B1 preferentially loads miRNAs carrying a GGAG tetra-nucleotide motif in their 3′ region into intraluminal vesicles [15]. Post-translational sumoylation of hnRNPA2B1 modulates its miRNA-binding affinity; specifically, by analogy with observations in activated immune cells, this modification selectively enriches pro-tumorigenic miRNA species in secreted exosomes [15,16,17]. This mechanosensitive sorting bias thus provides a plausible mechanism by which ECM stiffening in desmoplastic tumors progressively enriches the paracrine oncomiR load delivered to cancer cells. An additional loading mechanism involves YBX1, which contributes to the sorting of specific miRNAs into exosomes [18].

### 2.3. Exosome Uptake and TME Retention

Exosome internalization by recipient cells proceeds through several mechanisms, including macropinocytosis, endocytosis (clathrin- or caveolin-mediated), and direct fusion with the plasma membrane [19]. Real-time imaging using high-resolution live-cell imaging and confocal microscopy has validated these processes, confirming that the specific uptake pathway is highly dependent on the recipient cell type and surface protein interactions [20,21]. Notably, exosomal surface Integrins determine organotropic distribution: for example, exosomes carrying αvβ5 integrins tend to home to hepatic stroma, whereas those enriched in α6β4 and α6β1 preferentially accumulate in pulmonary tissue [22]. Within the desmoplastic tumor microenvironment, CAF-derived exosomes often bear surface fibronectin and tenascin-C, which promote their retention within the fibrotic extracellular matrix. This creates a locally concentrated paracrine reservoir of miRNAs that sustains chronic signaling to adjacent tumor cells, a phenomenon with important implications for both stromal biology and the limited penetration of chemotherapeutic agents.

## 3. Exosomal miRNAs in Desmoplasia and Stromal Remodeling

**Figure 1:**
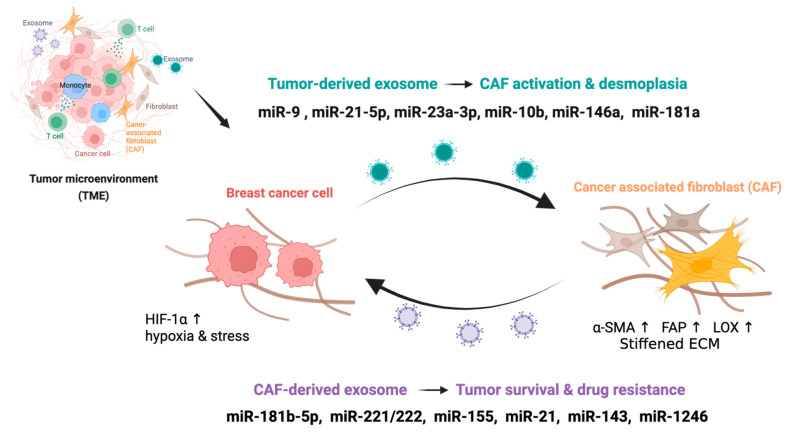
Exosomal miRNA crosstalk between breast cancer cells and CAFs. Breast cancer cells under hypoxic stress (HIF-1α↑) release exosomes containing miRNAs (miR-9, miR-21-5p, miR-23a-3p, miR-10b, miR-146a, miR-181a) that activate CAFs and promote desmoplasia (α-SMA↑, FAP↑, LOX↑, stiffened ECM). Reciprocally, CAF-derived exosomes deliver miRNAs (miR-181b-5p, miR-221/222, miR-155, miR-21, miR-143, miR-1246) back to tumor cells, enhancing survival and drug resistance. This bidirectional exosomal loop sustains the desmoplastic microenvironment and reinforces chemoresistance. Upward arrows (↑) indicate elevated expression levels.

Transformation of the quiescent breast stroma into a pro-tumorigenic, fibrotic niche depends on sustained intercellular reprogramming, with exosomal miRNAs serving as key molecular mediators of this process (Figure 1).

### 3.1. CAF Activation and Fibroblast Reprogramming

The transition from quiescent resident fibroblasts to activated cancer-associated fibroblasts (CAFs) represents the defining cellular event in the desmoplastic response. Tumor-derived exosomal miR-9 and miR-146a are among the earliest inducers of this phenotypic switch, promoting myofibroblastic differentiation marked by upregulation of α-SMA, fibroblast activation protein (FAP), and increased collagen secretion [8,23,24]. Exosomal miR-21-5p amplifies this process by suppressing PTEN in recipient fibroblasts, thereby activating PI3K/AKT signaling and driving autocrine TGF-β1 secretion, which sustains CAF activation. Once established, CAFs release exosomes enriched in miR-21, miR-143, and miR-155 back to tumor cells, completing a bidirectional feedback loop that underpins persistent desmoplasia and promotes an aggressive, stem-like phenotype in the cancer cells [25,26] (Table 1). Exosomal miR-9 further reprograms stromal fibroblasts by downregulating E-cadherin and STAT5B, reinforcing a pro-invasive, matrix-remodeling CAF phenotype [26]. The resulting paracrine TGF-β amplification loop not only perpetuates CAF activation but also promotes epithelial–mesenchymal transition (EMT) in adjacent cancer cells, a convergence particularly pronounced in triple-negative breast cancer [25,26] (Table 1).

### 3.2. ECM Remodeling and Mechanical Stiffening

Desmoplasia is defined by the pathological deposition and cross-linking of collagen I and III, fibronectin, and hyaluronan, which increases tissue stiffness from the 0.1–2 kPa range typical of normal mammary stroma to 10–40 kPa in desmoplastic tumors. Exosomal miR-181a, acting downstream of TGF-β signaling, stimulates fibroblasts to upregulate collagen production and lysyl oxidase (LOX) activity—the enzyme responsible for collagen cross-linking that drives ECM stiffening [34,35]. The resulting rigid matrix activates integrin-FAK-Rho/ROCK mechanotransduction in cancer cells, further enhancing exosome secretion and creating a positive mechanochemical feedback loop. Exosomal miR-23a-3p, released by hypoxic tumor cells, suppresses VHL and PTEN in recipient CAFs, leading to HIF-1α stabilization and increased LOX-mediated cross-linking [36]. Metastatic breast cancer cells (such as MDA-MB-231) actively secrete exosomes enriched with miR-10b, a process promoted by nSMase2 or ceramide. Once taken up by recipient non-malignant mammary epithelial cells, this exosomal miR-10b suppresses the protein levels of its target genes, HOXD10 and KLF4, thereby inducing an invasive phenotype in previously non-invasive cells [32].

### 3.3. Preparation of the Pre-Metastatic Niche

The miR-181 family comprises four mature paralogs—miR-181a, miR-181b, miR-181c, and miR-181d—each with distinct 5p/3p arm processing [29]. In breast cancer, miR-181a-5p, miR-181b-5p, and miR-181d-5p are the predominant oncogenic isoforms enriched in desmoplastic exosomes; their specific molecular targets and contributions to resistance are detailed in Section 4. Collectively, members of this family help shape distant stromal microenvironments to support metastatic colonization. Under hypoxic or genotoxic stress conditions, these miRNAs are packed into exosomes, where they suppress motility inhibitors in target cells and create a pro-inflammatory environment at future metastatic sites. Consequently, circulating exosomal miR-181a-5p serves as a real time indicator of systemic immunosuppressive priming. This highlights the clinical value of serial liquid biopsy monitoring, as rising plasma levels can signal pre-metastatic niche conditioning weeks before lesions become radiographically detectable [29].

### 3.4. Stromal Metabolic Reprogramming: The Reverse Warburg Effect

Exosomal miRNAs also orchestrate a metabolic symbiosis between tumor cells and desmoplastic CAFs. In this context, miR-181a-5p has been reported to silence NDRG2 in stromal cells, a mechanism linked to upregulated glycolytic activity and increased lactate and pyruvate secretion [33]; however, these data were derived from breast cancer epithelial cell line models rather than from CAF co-culture or stromal compartment systems, and extrapolation of the miR-181a-5p/NDRG2 glycolytic axis to desmoplastic CAF biology specifically remains inferential. The broader NDRG2-glycolysis connection is nonetheless consistent with CAF metabolic reprogramming associated with the reverse Warburg phenotype in breast cancer. Neighboring tumor cells import and oxidize these metabolites through mitochondrial oxidative phosphorylation, a phenomenon known as the reverse Warburg effect, enabling sustained proliferation even under nutrient-restricted conditions [33,37].

## 4. Exosomal miRNA-Driven Resistance: Senescence Evasion, Endocrine Escape, DNA Repair Impairment, EMT Promotion, and HER2 Therapy Bypass

Exosomal miRNA transfer between tumor cells and the desmoplastic stroma drives resistance to chemotherapy, endocrine therapy, and targeted agents through molecularly distinct but functionally convergent mechanisms (Figure 1).

### 4.1. The miR-181b-5p/BCLAF1/p53-p21 Axis: Senescence Evasion

Desmoplastic CAFs subvert anthracycline-induced senescence through a paracrine exosomal axis centered on miR-181b-5p. Cellular senescence, a p53/p21-dependent form of permanent growth arrest, is one of the principal cytotoxic responses to anthracyclines such as doxorubicin, and its evasion is a mechanistically distinct route to chemoresistance that is separate from classical apoptosis blockade [30]. CAF-secreted exosomes are enriched in miR-181b-5p under doxorubicin pressure, and their transfer to recipient tumor cells confers a drug-resistant phenotype by directly suppressing BCLAF1 (BCL2-associated transcription factor 1), a transcriptional co-activator required for p53 stabilization [30]. Loss of BCLAF1 attenuates p53 accumulation, preventing the induction of p21-mediated G1 arrest and allowing continued cell cycling despite unrepaired DNA damage (Table 2). Importantly, this mechanism operates in both luminal and triple-negative breast cancer contexts, reflecting the broad permissiveness of the exosomal delivery route across receptor subtypes. Beyond senescence evasion, BCLAF1 suppression amplifies autocrine TGF-β signaling in tumor cells, reinforcing CAF activation and collagen deposition in a feedforward loop that simultaneously upregulates mesenchymal gene programs, including ABC-family drug efflux transporters, further reducing intracellular anthracycline accumulation [30]. This dual consequence (senescence evasion plus efflux upregulation) makes the miR-181b-5p/BCLAF1 axis a mechanistically layered resistance node. Consistent with this, pharmacological inhibition of exo-miR-181b-5p in preclinical models restores tumoral BCLAF1, re-engages p53/p21-dependent senescence, and improves doxorubicin efficacy in vivo, establishing therapeutic proof-of-concept for targeting this axis [30].

### 4.2. The miR-221/222/ESR1-p27 Axis: Endocrine Therapy Resistance

Resistance to endocrine therapies such as tamoxifen in ER+/PR+ breast cancer is often driven by reduced expression of ERα and a loss of cell-cycle checkpoint control. Exosomal miR-221 and miR-222, secreted by both tumor cells and cancer-associated fibroblasts (CAFs), play a direct role in this process by targeting ESR1 (which encodes ERα) and CDKN1B (which encodes the cell-cycle inhibitor p27Kip1) in recipient cells [38]. Downregulation of ESR1 renders cells insensitive to estrogen deprivation, while loss of p27 abrogates the G1 arrest that is required for tamoxifen to effectively block proliferation (Table 2). Together, these effects enable sustained cell cycling even in the presence of hormonal blockade. Importantly, direct transfer of miR-221/222 via exosomes from resistant to sensitive cells has been shown to transmit this resistant phenotype, offering a mechanism for the intercellular spread of endocrine resistance within a tumor. Furthermore, miR-221/222 also confers resistance to trastuzumab-based chemotherapy, broadening the clinical impact of this pathway [38,39].

### 4.3. The miR-155/RAD51-FOXO3a Axis: DNA Repair Impairment in TNBC

In triple-negative breast cancer (TNBC), exosomal miR-155 acts as a potent modulator of therapeutic response through three distinct axes. First, it directly represses RAD51, a central component of high-fidelity homologous recombination (HR) repair. While this loss of repair capacity initially sensitizes cells to DNA-damaging agents and radiation, the resulting genomic instability can drive long-term resistance by facilitating the selection of aggressive, multidrug-resistant subclones. Second, miR-155 targets the transcription factor FOXO3a, thereby relieving its inhibitory control over cancer stem cell (CSC) maintenance and expanding the self-renewing, drug-tolerant cell population [31]. Finally, by delivering miR-155 to the tumor microenvironment, cancer cells suppress SHIP1 in tumor-associated macrophages [41]. This reprogramming creates an immunosuppressive milieu that potentially limits the efficacy of immune checkpoint inhibitors in TNBC patients [27,31,41].

### 4.4. CAF-Derived Exosomes and the miR-181d-5p/CDX2/HOXA5 Axis: Promotion of EMT and Metastasis

Within the breast tumor microenvironment, cancer-associated fibroblasts (CAFs) actively secrete exosomes rich in miR-181d-5p, which modulate the behavior of adjacent cancer cells. Following internalization, exosomal miR-181d-5p directly targets and suppresses the transcription factor CDX2 [40]. Under normal conditions, CDX2 binds to the HOXA5 promoter to sustain its expression and restrain oncogenic signaling. However, miR-181d-5p-mediated silencing of CDX2 results in marked downregulation of HOXA5—a critical checkpoint in tumor progression. Disruption of the CDX2/HOXA5 axis initiates a cascade of phenotypic changes that promote aggressive disease. Mechanistically, HOXA5 loss induces the epithelial–mesenchymal transition (EMT), characterized by reduced E-cadherin expression and concurrent upregulation of mesenchymal markers including Vimentin, N-cadherin, and Snail [40] (Table 2). This transition substantially enhances the migratory and invasive capacity of breast cancer cells. Moreover, by suppressing HOXA5-mediated apoptotic signaling, this exosomal crosstalk supports cancer cell survival during systemic dissemination. These findings identify a key mechanism through which CAF-derived exosomes remodel the desmoplastic tumor microenvironment to drive metastasis and therapeutic resistance in breast cancer.

### 4.5. The miR-1246/AXNA6-BAK1 Axis: Targeted Therapy Resistance in HER2+ Disease

In HER2-amplified breast cancer, exosomal miR-1246 contributes to resistance against both trastuzumab and lapatinib through multiple mechanisms. It directly suppresses Annexin A6 (ANXA6) and the pro-apoptotic regulator BAK1, thereby inhibiting mitochondrial apoptosis even when HER2 signaling is pharmacologically blocked [28] (Table 2). In parallel, miR-1246-mediated downregulation of PTEN sustains constitutive PI3K/AKT signaling downstream of HER2, providing an alternative survival pathway that bypasses the intended effects of HER2-targeted therapy. Clinically, rising levels of circulating exosomal miR-1246 have been observed in patients receiving trastuzumab as they transition to a drug-resistant state. This association suggests that exosomal miR-1246 may serve as an early pharmacodynamic indicator of treatment failure, offering potential utility in monitoring response to HER2-targeted therapies.

## 5. Convergence on Shared Signaling Pathways

Although the resistance mechanisms described above involve distinct miRNA-target interactions, they converge on a limited set of core signaling nodes. This functional convergence amplifies the biological impact of exosomal miRNA signaling and helps explain the breadth of drug resistance phenotypes observed in the clinical setting.

### 5.1. PTEN/PI3K/AKT/mTOR

A common point of convergence among several exosomal miRNAs, including miR-181a, miR-221, miR-21-5p, and miR-1246, is the suppression of PTEN. Loss of PTEN leads to constitutive activation of AKT, which in turn drives multiple pro-survival and drug-resistant phenotypes [33,39,42]. First, AKT signaling upregulates ABC efflux transporters such as ABCB1 (P-gp) and ABCG2, reducing intracellular accumulation of chemotherapeutic agents [43]. Second, AKT-mediated phosphorylation of FOXO3a results in its nuclear exclusion and transcriptional inactivation, thereby suppressing expression of the pro-apoptotic factors BIM and PUMA [44]. Third, sustained AKT activity activates mTORC1, promoting translation of pro-survival effectors including cyclin D1, HIF-1α, and MCL-1. Beyond its cell-autonomous effects, mTOR activity in cancer-associated fibroblasts (CAFs) has been shown to enhance exosome secretion [45]. This creates a positive feedback loop in which desmoplastic stiffness and exosome production reinforce one another, further amplifying the signaling network that drives therapeutic resistance.

### 5.2. EMT and Acquisition of Invasive, Drug-Tolerant Phenotypes

Exosomal miR-181a and miR-9 contribute to epithelial–mesenchymal transition (EMT) by suppressing E-cadherin and activating ZEB1/2 and SNAIL-family transcription factors, resulting in reduced intercellular adhesion and increased migratory capacity [24,35]. EMT is a prerequisite for invasion through the stiffened desmoplastic extracellular matrix and is associated with broad-spectrum resistance to cytotoxic agents. It also reduces MHC-I surface expression, potentially limiting immune recognition. In triple-negative breast cancer (TNBC), the physical effects of ECM stiffness and exosomal signaling converge to amplify drug resistance. Mechanical stiffness drives YAP/TAZ activation, while exosomal miR-155-mediated suppression of LATS2 further promotes nuclear accumulation of YAP [31]. Nuclear YAP directly transactivates multiple resistance-related genes, including ABCB1, MCL-1, and survivin (BIRC5), thereby providing a mechanistic link between the desmoplastic tumor microenvironment and the acquisition of molecular drug resistance.

### 5.3. YAP/TAZ Mechanotransduction

YAP and its paralog TAZ function as primary mechanotransducers, relaying signals from the extracellular matrix (ECM) to the nucleus in response to changes in tissue stiffness [46]. Under conditions of increased matrix stiffness, nuclear accumulation of YAP/TAZ drives transcription of ECM components and matricellular proteins such as CTGF, CYR61, and ANKRD1. Their upregulation further reinforces stromal stiffening, establishing a self-sustaining mechanochemical feedback loop that amplifies desmoplasia [46,47]. Exosomal miR-155 can uncouple YAP activity from this mechanical context by disrupting LATS2-mediated inhibitory phosphorylation of YAP. This results in constitutive nuclear YAP localization and sustained transcriptional activity, even in the absence of high matrix stiffness. Consequently, drug-resistant gene expression programs become embedded across the tumor independently of local mechanical cues, broadening the impact of YAP-driven resistance mechanisms [31,46,47].

## 6. Circulating Exosomal miRNAs as Liquid Biopsy Biomarkers

### 6.1. Rationale and Advantages over Conventional Liquid Biopsy

Standard liquid biopsy approaches, including the analysis of cell-free DNA (cfDNA) and total circulating miRNAs, are limited by the susceptibility of naked nucleic acids to RNase-mediated degradation in plasma. In contrast, exosomal miRNAs are encapsulated within a protective lipid bilayer, which confers remarkable stability during prolonged storage and across repeated freeze-thaw cycles in biofluids such as plasma, serum, urine, and breast milk [48]. Equally important is the biological origin of exosomes. They are actively secreted by viable cells rather than released passively from necrotic or apoptotic debris. As a result, their miRNA content provides a real-time functional snapshot of both the tumor and its surrounding stroma. This dynamic information is not readily obtainable from tissue biopsies, which are constrained by spatial sampling bias and cannot be serially collected for longitudinal treatment monitoring [49]. It should be noted, however, that the clinical application of exosomal miRNA liquid biopsy remains in an exploratory phase; the studies supporting these advantages are largely preliminary, and prospective clinical validation in large, diverse cohorts is required before any diagnostic or monitoring utility can be considered established.

### 6.2. Predicting Pathological Complete Response to Neoadjuvant Chemotherapy

Pathological complete response (pCR) to neoadjuvant chemotherapy (NAC) serves as the primary surrogate endpoint for long-term outcomes in both triple-negative and HER2-positive breast cancer. Preliminary evidence from exploratory studies suggests that pre-treatment levels of circulating exosomal miRNAs, particularly miR-181b-5p and miR-21-5p, may carry predictive associations with pCR, with elevated baseline concentrations correlating with reduced pCR rates in small, unvalidated cohorts [50,51]. Beyond baseline prediction, serial monitoring of these exosomal miRNA levels during NAC has been proposed as a means of obtaining early pharmacodynamic evidence of treatment failure, with signals hypothesized to be detectable before surgical restaging; however, this application remains entirely exploratory and has not yet been validated in prospective clinical trials.

### 6.3. Monitoring Endocrine and Targeted Therapy Resistance

In luminal and HER2-positive breast cancer, serial profiling of exosomal miRNAs has been explored in preliminary studies as a non-invasive approach for monitoring the emergence of therapeutic resistance. In exploratory analyses, rising circulating levels of miR-221/222 during tamoxifen treatment have been reported to precede clinical evidence of endocrine failure by weeks to months, suggesting a potential early window for treatment escalation that requires prospective validation [52]. Similarly, in HER2-positive patients receiving trastuzumab, increasing exosomal miR-1246 and miR-155 levels have been reported in preliminary studies to associate with the transition to a resistant state [28]. Serial liquid biopsy holds potential practical advantages over tissue biopsy in this setting, including greater clinical feasibility and the ability to capture inter-lesional clonal heterogeneity; however, these theoretical advantages await confirmation through prospective clinical trials before serial exosomal miRNA profiling can be recommended as a clinically validated monitoring strategy.

### 6.4. Early Detection and Subtype Discrimination

Exosomal miRNA panels are currently under active investigation as tools for early breast cancer detection and molecular subtype discrimination; all findings to date are preliminary and await prospective clinical validation. A panel consisting of miR-335, miR-628, and miR-422a shown preliminary discriminatory value in plasma-based assays for molecular subtype classification, distinguishing TNBC from HER2-positive breast cancer with a sensitivity of 68% and specificity of 81% in a large prospective cohort of 435 patients [53]. Broader multi-miRNA signatures have also shown promise for further stratifying TNBC. Combinations that include miR-21-5p, miR-155-5p, and miR-181b-5p have achieved area under the curve (AUC) values in the range of 0.85–0.92 for discriminating high-desmoplasia from low-desmoplasia TNBC in exploratory cohort studies [54,55,56], with independent prognostic value for disease-free survival demonstrated in multivariate analyses, although prospective validation in ancestrally diverse cohorts remains needed before these signatures can be considered clinically actionable [57].

### 6.5. Standardization and Technical Considerations

Despite compelling preliminary evidence, the clinical implementation of exosomal miRNA biomarkers remains constrained by substantial methodological heterogeneity. Available isolation platforms, including differential ultracentrifugation, density gradient separation, size-exclusion chromatography, precipitation reagents, and immunoaffinity capture, yield preparations that differ significantly in vesicle purity, composition, and miRNA recovery. This variability limits cross-laboratory comparability and hinders validation efforts [20]. Similarly, normalization strategies for qRT-PCR quantification, such as cel-miR-39 spike-ins, miR-16-5p, and U6 snRNA, each carry known limitations when applied in the exosomal context. Efforts to address these challenges are underway. The International Society for Extracellular Vesicles (ISEV) MISEV2023 reporting guidelines, together with the EQUATOR network, provide minimum standards for EV isolation, characterization (including nanoparticle tracking analysis, electron microscopy, and tetraspanin profiling), and miRNA quantification [20]. Crucially, strict adherence to these standards within prospective biomarker studies is an essential prerequisite for generating the robust, reproducible data required for the successful transition of EV-miRNA panels into regulated clinical practice [20]. Looking ahead, clinical platforms will likely integrate exosomal miRNA panels with complementary modalities such as circulating tumor DNA (ctDNA) analysis and EV proteomics. When interpreted through machine learning algorithms, these multi-dimensional data could provide a real-time, comprehensive assessment of desmoplastic burden and resistance status from a single blood draw [58,59].

## 7. Therapeutic Strategies Targeting the Exosomal miRNA Axis

### 7.1. Rationale for Targeting the Desmoplastic miRNA Network

The therapeutic implications of exosomal miRNA biology in desmoplastic breast cancer can be considered in three categories. First, blocking the delivery of oncogenic miRNAs (oncomiRs) from cancer-associated fibroblasts (CAFs) to tumor cells may restore apoptotic sensitivity by de-repressing key effectors including BCLAF1, the p53/p21 axis, and BAX [60]. Second, re-introducing tumor-suppressive miRNAs that are depleted during desmoplastic reprogramming could normalize the CAF phenotype and reduce extracellular matrix stiffness—an approach referred to as stromal normalization [61]. Third, directly inhibiting exosome biogenesis or secretion has the potential to attenuate the entire pro-desmoplastic signaling network [61]. However, given the functional redundancy and feedback architecture inherent to these pathways, it is unlikely that any single intervention will be sufficient. Combination strategies designed to target multiple nodes simultaneously will likely be required to durably overcome resistance.

### 7.2. Anti-miRNA Oligonucleotides (AntagomiRs)

Chemically modified antisense oligonucleotides, known as antagomiRs, represent the most clinically advanced class of miRNA-targeted therapeutics. These agents function by sequestering specific oncomiRs, thereby restoring expression of their suppressed targets. Several examples illustrate their potential in preclinical models. Anti-miR-181b-5p treatment in CAF-tumor co-culture systems restores BCLAF1 expression, stabilizes p53, and increases apoptotic sensitivity to doxorubicin by 2- to 4-fold [30]. The feasibility of antagomirs for in vivo miRNA silencing in breast tumors was established by Ma et al. (2010), who showed that systemic cholesterol-conjugated anti-miR-10b antagomirs markedly suppressed lung metastasis in 4T1 mammary tumor models without primary tumor toxicity [62]. Extending this principle, locked nucleic acid (LNA)-modified anti-miR-21 constructs have been shown to suppress PTEN downregulation, reduce CAF activation, and restore paclitaxel sensitivity in TNBC xenograft models [63]. Anti-miR-221 similarly recovers p27 and ERα expression, reversing tamoxifen resistance in ER-positive models [52,64]. Clinical translation of these agents requires efficient delivery to the tumor microenvironment. This challenge is being addressed through encapsulation in lipid nanoparticles (LNPs), PLGA nanoparticles, or cyclodextrin-based formulations, which improve stromal penetration and reduce systemic exposure [65].

### 7.3. Exosome Biogenesis and Secretion Inhibitors

Rather than targeting individual miRNAs, an alternative strategy involves inhibiting exosome production to globally attenuate the secretion of oncogenic miRNAs. GW4869, a pharmacological inhibitor of the ceramide-dependent nSMase2 pathway responsible for intraluminal vesicle (ILV) formation, reduces exosome secretion and has been shown to attenuate metastatic spread and stromal remodeling in multiple tumor models, establishing the broader proof-of-concept that nSMase2 blockade can disrupt oncogenic exosomal miRNA signaling [66,67]; its application in breast cancer is under active preclinical investigation. More targeted genetic inhibition has also demonstrated efficacy. Rab27a silencing via LNP-delivered siRNA selectively reduces fusion of multivesicular bodies (MVBs) with the plasma membrane, decreasing plasma exosomal miR-21-5p levels by over 70% and attenuating CAF activation in desmoplastic TNBC patient-derived organoid co-cultures. Interfering with exosome uptake represents another point of intervention [68]. Dimethyl amiloride (DMA), which inhibits macropinocytosis-mediated exosome internalization by recipient cells, has demonstrated synergistic effects with paclitaxel in preclinical studies [69].

### 7.4. Engineered Exosomes for Precision Delivery

The inherent cell-type tropism and low immunogenicity of exosomes have positioned them as promising vehicles for therapeutic delivery. In the context of TNBC, surface-functionalized exosomes—decorated with peptides targeting cancer-associated fibroblasts (CAFs) (e.g., anti-FAP or anti-alpha-SMA) and loaded with miR-143 mimics or anti-miR-181b antogomiR—enable selective CAF uptake. This targeted approach reduces alpha-SMA expression, decreases collagen secretion, and promotes the reversion of primary TNBC CAFs to a quiescent state [70]. Alternatively, engineering tumor-derived exosomes to endogenously carry tumor-suppressive miRNAs leverages their natural stromal tropism [71,72]. This strategy bypasses the need for synthetic vectors and may offer reduced immunogenicity compared to conventional lipid-based delivery systems.

### 7.5. Regulatory and Manufacturing Considerations

The clinical translation of exosomal miRNA therapeutics is contingent upon addressing several interrelated manufacturing and regulatory challenges. For engineered exosomes intended for therapeutic use, production must adhere to Good Manufacturing Practice (GMP) standards, ensuring validated consistency in cargo loading, defined surface marker profiles, and rigorous safety evaluations to preclude off-target immunostimulation or unintended nucleic acid transfer [73,74,75]. Concurrently, the development of companion diagnostic assays for patient stratification requires the establishment of both analytical validity (ensuring reproducible and reliable measurement) and clinical validity, which confirms the assay’s accuracy in predicting therapeutic benefit. Given the integrated nature of this therapeutic-diagnostic modality, early and strategic engagement with regulatory bodies such as the FDA and EMA through pre-IND consultations is essential to establish clear and coordinated approval pathways.

## 8. Health Disparities and the Exosomal miRNA Landscape

Triple-negative breast cancer (TNBC) disproportionately affects women of African ancestry, who present with higher incidence rates, younger age at diagnosis, more aggressive histopathology, and significantly worse survival outcomes compared to women of European ancestry [76]. These disparities are believed to stem from biological differences inherent to the tumors themselves. Emerging evidence suggests that exosomal miRNA profiles differ systematically between ancestral groups in clinically meaningful ways. Specifically, women of African ancestry with TNBC exhibit elevated levels of plasma exosomal miR-21-5p and miR-181b-5p relative to stage-matched non-Hispanic White patients. These expression differences correlate with higher desmoplasia scores on histopathological assessment and lower rates of pathologic complete response (pCR) to standard neoadjuvant chemotherapy (NAC) regimens [77,78].

Whether these differences stem from inherited variation in miRNA biogenesis or cargo-sorting machinery, ancestral variation in hormonal or metabolic regulation of CAF activity, or differential epigenetic silencing of tumor-suppressive miRNA loci remains under active investigation.

## 9. Challenges and Future Directions

### 9.1. CAF Subtype Heterogeneity and Spatial miRNA Profiling

The exosomal miRNA secretomes of distinct cancer-associated fibroblast (CAF) subtypes, namely myofibroblastic (myCAF), inflammatory (iCAF), and antigen-presenting (apCAF), remain incompletely characterized, yet they are likely to contribute differentially to specific axes of therapy resistance [79]. A more comprehensive understanding of this heterogeneity will require the integration of single-cell RNA sequencing with spatial transcriptomics and extracellular vesicle (EV) proteomics. Such multimodal approaches are essential to resolve which CAF populations are responsible for particular oncomiR signals and to identify subtype-selective intervention targets that preserve CAF subsets with homeostatic or immunostimulatory functions.

### 9.2. Integration with Immunotherapy

Understanding the intersection of exosomal miRNA signaling and the immune tumor microenvironment (TME) has acquired clinical urgency, given the incorporation of immune checkpoint inhibitors such as atezolizumab and pembrolizumab into frontline TNBC regimens. The desmoplastic extracellular matrix (ECM) characteristic of TNBC creates physical barriers that limit cytotoxic T-lymphocyte infiltration. Elucidating how anti-desmoplastic miRNA interventions modulate PD-L1/PD-1 checkpoint efficacy will therefore be essential for designing rational combination strategies that both normalize the stromal architecture and restore durable anti-tumor immune responses [80,81].

### 9.3. Machine Learning and Multi-Omics Integration

Intratumoral clonal heterogeneity poses significant challenges to diagnostics. Because multiple subclones may simultaneously release distinct exosomal populations with unique miRNA profiles, they generate a composite circulating signal in the blood sample that single-marker tests cannot decode. To address this complexity, researchers in the field have begun exploring how machine learning models that integrate exosomal miRNA panels with complementary data streams, such as circulating tumor DNA (ctDNA), EV proteomics, and serial imaging. Although still in the research phase, this multi-dimensional approach aims to extract significantly more prognostic information from a single blood draw than current static methods allow [27,82]. However, the development and rigorous validation of such models will require large, prospective cohorts with densely sampled serial specimens collected at standardized timepoints throughout treatment [83].

### 9.4. Longitudinal Dynamics and Therapeutic Windows

While we know exosomal miRNAs drive everything from early CAF activation to drug resistance, most studies have focused on a single snapshot at a specific time point. To better understand this progression, we need longitudinal studies that pair regular blood tests with imaging like MR or ultrasound elastography [84]. This combined approach would enable us to pinpoint the exact moment at which resistance emerges and to identify the optimal “therapeutic window”, which is often overlooked in static studies.

## 10. Conclusions

Exosomal miRNAs function as active molecular architects of the desmoplastic breast cancer microenvironment, rather than serving merely as passive disease markers. Through bidirectional intercellular transfer between cancer cells and CAFs, they orchestrate the fibrotic ECM remodeling, metabolic reprogramming, and mechanical stiffening that define desmoplasia. Concurrently, these exosomal signals program multi-layered treatment resistance through distinct yet convergent axes: miR-181b-5p/BCLAF1/p53-p21 drives senescence evasion; miR-221/222/ESR1-p27 facilitates endocrine escape; miR-155/RAD51-FOXO3a impairs DNA repair and expands the cancer stem cell (CSC) population; miR-181a/BAX-Bim blocks mitochondrial apoptosis; and miR-1246/AXNA6-BAK1 confers targeted therapy resistance in HER2+ disease. Despite their diversity, these pathways converge on central signaling hubs, including the PTEN/PI3K/AKT axis, Wnt/β-catenin and Notch stemness programs, and YAP/TAZ mechanotransduction, that collectively sustain a chemoresistant, pro-desmoplastic phenotype.

As circulating biomarkers, exosomal miRNA panels offer clinically actionable information on desmoplastic burden, response to neoadjuvant chemotherapy, and the early emergence of endocrine or targeted therapy resistance, information that serial tissue biopsy cannot feasibly provide. Realizing this potential, however, will require the establishment of consensus on isolation and quantification standards, prospective validation in ancestrally diverse patient populations, and integration with complementary liquid biopsy modalities.

Therapeutically, several mechanistically rational strategies hold promise for disrupting the desmoplastic miRNA loop and restoring drug sensitivity. These strategies include antagomirs, miRNA replacement therapy, exosome biogenesis inhibitors, and engineered exosome delivery platforms. However, translating these approaches into clinical practice requires substantial progress in areas such as TME-targeted delivery, mitigation of immunogenicity, and the development of scalable GMP manufacturing pipelines. The exosomal miRNA axis represents one of the most compelling and underexplored opportunities in breast cancer biology. Therapeutics designed to systematically target this axis hold genuine potential to improve treatment outcomes in the most treatment-resistant types of breast cancer.

## Figures and Tables

**Table 1:** Subtype-specific exosomal miRNA signatures and clinical consequences in breast cancer.

BC Subtype	Key Exosomal miRNAs	Primary Targets	Clinical Consequence	References
TNBC	miR-155-5p, miR-181a-5p, miR-1246, miR-9-5p	BAX, RAD51, PTEN, E-cadherin	EMT and aggressive spread; resistance to anthracyclines and taxanes	[27,28]
Luminal (ER+/PR+)	miR-221-3p, miR-222-3p, miR-181b-5p, miR-21-5p	ESR1, p27 (CDKN1B), BCLAF1, PTEN	Escape from tamoxifen; CDK4/6 inhibitor resistance	[29,30]
HER2+	miR-181a-5p, miR-1246, miR-155-5p	ATM, PTEN, AXNA6, BAK1	Sustained PI3K/AKT signaling; resistance to trastuzumab and lapatinib	[27,28]
BRCA1-mutant TNBC	miR-155-5p, miR-182-5p	BRCA1, FOXO3a, RAD51	Faulty DNA repair; PARP inhibitor resistance	[31]
Inflammatory BC (IBC)	miR-181a-5p, miR-10b-5p	HOXD10, TIAM1, CDH1	Spread through lymph vessels; skin metastasis	[32,33]

Key exosomal miRNA species found in each major breast cancer subtype, the genes they target, the clinical consequences of their activity, and key published references. BC = breast cancer; CAF = cancer-associated fibroblast; EMT = epithelial–mesenchymal transition; IBC = inflammatory breast cancer; MDSC = myeloid-derived suppressor cell; TNBC = triple-negative breast cancer.

**Table 2:** Key miRNA-driven pathways that cause treatment resistance in breast cancer.

miRNA/Target Axis	BC Subtype	Molecular Target (s)	Resistance Mechanism	Reference
miR-181b-5p/BCLAF1	Luminal BC, TNBC	BCLAF1 → reduced p53/p21	Abrogates therapy-induced senescence; sustains TGF-beta feedforward loop	[30]
miR-221-3p/222-3p/p27-ESR1	Luminal (ER+)	ESR1, CDKN1B (p27), PUMA	Downregulates ERalpha, eliminating estrogen-dependence; loss of p27 removes the G1 checkpoint tamoxifen requires	[38,39]
miR-155-5p/RAD51-FOXO3a	TNBC	RAD51, FOXO3a, SHIP1	Impairs HR-mediated DSB repair; expands CSC population; drives platinum/taxane resistance	[31]
miR-181d-5p/ CDX2/HOXA5	All subtypes	CDX2/HOXA5	Promote EMT by regulating CDX2/HOXA5	[40]
miR-1246/AXNA6-BAK1	HER2+, metastatic BC	Annexin A6, BAK1	Spread through lymph vessels; skin metastasis	[28]

Summary of the major exosomal miRNA resistance pathways, showing the miRNA involved, which breast cancer subtype is affected, the genes that are silenced, how resistance develops, and key references. BC = breast cancer; CAF = cancer-associated fibroblast; CSC = cancer stem cell; HR = homologous recombination; TNBC = triple-negative breast cancer.

## Data Availability

No new data were created or analyzed in this study. Data sharing is not applicable.
